# Association between depression and cognitive function in middle-aged and older adults: a study based on the health and retirement study

**DOI:** 10.3389/fpsyg.2026.1811924

**Published:** 2026-05-07

**Authors:** Yang Zhao, Leyao Miao, Hongji Qin, Tao Jiang, Zhibin Chen, Lei Zhang, Na Wang

**Affiliations:** 1 School of Humanities and Management, Guilin Medical University, Guilin, China; 2 School of Public Health, Fujian Medical University, Fuzhou, China; 3 The Second School of Clinical Medicine, Guilin Medical University, Guilin, China; 4 Guilin Social Welfare Hospital (Guilin Mental Health Center), Guilin, China; 5 School of Artificial Intelligence Medicine, Guilin Medical University, Guilin, China; 6 Guangxi Key Laboratory of Environmental Exposomics and Entire Lifecycle Health, Guangxi Key Laboratory of Diabetic Systems Medicine, School of Public Health, Guilin Medical University, Guilin, China

**Keywords:** aged, anxiety, cognitive dysfunction, depression, HRS, mediation analysis

## Abstract

**Objective:**

To describe the distribution of depression and cognitive function status among adults aged 50 years and older, identify factors associated with cognitive function status, and examine the association between depression and cognitive function as well as the potential mediating roles of anxiety, health status, and eyesight.

**Methods:**

Cross-sectional data from 3,434 participants aged 50 years and older in the 2022 Health and Retirement Study were analyzed. Cognitive function was assessed using the TICS-27, depression using the 8-item CES-D, and anxiety using the 5-item BAI. Univariate analyses, multivariate ordered logistic regression, and mediation analyses were performed.

**Results:**

Among participants, 83.8% were cognitively normal, 13.7% had cognitive impairment no dementia (CIND), and 2.4% had dementia. Ordered logistic regression showed that age, race, educational level, marital status, total household income, eyesight, BMI category, and depression were significantly associated with cognitive function status. Compared with participants aged 50–59 years, those aged 70–79 years and ≥80 years had higher odds of being in a worse cognitive function category (OR = 2.156 and 3.776, respectively). Depressed participants were more likely to have worse cognitive function status than non-depressed participants (OR = 1.990). Anxiety, health status, and eyesight partially mediated the association between depression and cognitive function, accounting for 25.66, 32.19, and 15.19% of the total effect, respectively.

**Conclusion:**

Depression was significantly associated with worse cognitive function status among middle-aged and older adults, and anxiety, health status, and eyesight may partially explain this association. These findings highlight the importance of considering psychological, general health, and sensory factors in the early identification and comprehensive intervention of cognitive decline.

## 1 Introduction

Global population aging has become an increasingly prominent issue in the 21st century, posing important challenges to public health and social care systems worldwide. According to United Nations projections, the global population aged 60 years and older will reach 2.1 billion by the middle of this century, with China accounting for nearly one quarter of this population (Tang, 2022). According to the China Statistical Yearbook 2024, by the end of 2023, individuals aged 50 years and older accounted for 37.80% of the total population in China, among whom 216.76 million were aged 65 years and older, representing 15.4% of the total population (National Bureau of Statistics of China, 2024). Population aging has been accompanied by a growing burden of chronic non-communicable diseases such as hypertension, diabetes, and arthritis, as well as neurodegenerative disorders including Alzheimer’s disease and Parkinson’s disease, and mental health problems such as depression and anxiety (National Health Commission of the People’s Republic of China, 2022). Therefore, promoting healthy aging and addressing age-related health challenges have become important public health priorities in China (Xinhua News Agency, 2024).

Cognitive function refers to a range of conscious mental activities, including memory, calculation, orientation, language comprehension, and expression (Larner, 2008). With advancing age, cognitive function may gradually decline and further develop into cognitive impairment and even dementia (Chinese Expert Consensus Group on Prevention and Treatment of Cognitive Dysfunction, 2006). The World Health Organization estimated that there were approximately 55 million people living with dementia worldwide in 2019, and this number is projected to increase to 139 million by 2050 (Weidner, 2023). In China, a recent study reported that approximately 16.99 million people were living with Alzheimer’s disease and other dementias in 2021, and both prevalence and mortality increased with age (Wang et al., 2024). These findings highlight the importance of identifying factors associated with cognitive function in middle-aged and older adults in order to delay cognitive decline and improve quality of life.

Depression is one of the most common mental health problems among middle-aged and older adults (Lu et al., 2020). Depression, also referred to as depressive disorder, is a common mental disorder characterized by persistent low mood, psychomotor retardation, and reduced appetite (Kang and Cao, 2024; Liang et al., 2025). Previous studies have shown that depressive symptoms are closely associated with cognitive function and may be related to poorer cognitive outcomes in older adults (Ismail et al., 2017; Livingston et al., 2020; Lu et al., 2020; Su et al., 2023). At the same time, the association between depression and cognitive function may involve multiple factors. Late-life depression and anxiety often co-occur (Beekman et al., 2000), and anxiety has also been associated with subsequent dementia risk (Santabárbara et al., 2019). Poorer self-rated health has been associated with depressive symptoms (Peleg and Nudelman, 2021) as well as with incident dementia in older adults (Montlahuc et al., 2011). Impaired eyesight has likewise been linked to depression (Virgili et al., 2022) and cognitive decline (Nagarajan et al., 2022). Therefore, examining the association between depression and cognitive function from psychological, general health, and sensory perspectives has both theoretical and practical significance.

Although previous studies have examined the relationships among depression, anxiety, and cognitive outcomes, several gaps remain. Many studies have focused on specific regions or selected populations, examined only single outcomes such as mild cognitive impairment or dementia, or included a limited number of sociodemographic and health-related factors. In addition, the potential roles of anxiety, health status, and eyesight in the association between depression and cognitive function have not been sufficiently examined, especially in large population-based studies.

Using data from the 2022 Health and Retirement Study (HRS), the present study aimed to describe the distribution of depression and cognitive function status among middle-aged and older adults, identify factors associated with cognitive function status, and further examine the potential roles of anxiety, health status, and eyesight in the association between depression and cognitive function.

## 2 Materials and methods

### 2.1 Data source and participants

The data used in this study were derived from the 2022 Health and Retirement Study (HRS), a nationally representative longitudinal study of U.S. adults aged 50 years and older (Weden et al., 2018; Natale et al., 2022). The HRS began in 1992 and has conducted follow-up surveys every 2 years. It includes extensive information on demographic characteristics, socioeconomic status, health conditions, and cognitive function (Mezuk et al., 2011; Mitchell et al., 2022; Hanes and Clouston, 2023). The study was approved by the Institutional Review Board of the University of Michigan, and all participants provided informed consent (Mezuk et al., 2011). In the present study, participants aged 50 years and older who completed both the psychosocial questionnaire and the cognitive assessment in the 2022 HRS were eligible. After excluding respondents with missing data on key variables, including depression, anxiety, and cognitive function measures, a total of 3,434 participants were included in the final analysis.

### 2.2 Measures

#### 2.2.1 Outcome variable: cognitive function

Cognitive function was assessed using the HRS-modified Telephone Interview for Cognitive Status (TICS-27). This instrument was specifically adapted to better reflect neurophysiological decline and to reduce the influence of education and other sociocultural factors compared with items such as vocabulary (Karlamangla et al., 2009). The TICS-27 includes immediate and delayed word recall (10 points each), serial subtraction of 7 from 100 for five trials (5 points), and backward counting from 20 (2 points), yielding a total score ranging from 0 to 27 (Ghisletta et al., 2012). According to established HRS criteria (Crimmins et al., 2011), TICS-27 scores were classified into three categories: normal cognition (12–27), cognitive impairment no dementia (CIND, 7–11), and dementia (0–6). In the present study, cognitive function status was treated as an ordinal outcome variable, ordered from normal cognition to CIND and dementia. For descriptive purposes, participants classified as CIND or dementia were considered to have cognitive impairment overall. The Cronbach’s alpha coefficient of the TICS-27 in this sample was 0.660, indicating below the conventional acceptable threshold (≥0.70) and only moderate internal consistency, which may introduce some measurement error for cognitive classification.

#### 2.2.2 Predictor variables: depression and anxiety

Depressive symptoms were assessed using the 8-item Center for Epidemiologic Studies Depression Scale (CES-D) (Radloff, 1977). The items included feeling depressed, feeling that everything was an effort, restless sleep, feeling happy, feeling lonely, enjoying life, feeling sad, and being unable to get going. Participants responded to each item using a yes/no format (“yes” = 1, “no” = 0), and two positively worded items were reverse-coded. Item scores were summed to produce a total score ranging from 0 to 8, with higher scores indicating more severe depressive symptoms. Previous studies have shown that the 8-item CES-D has good internal consistency, with Cronbach’s alpha values ranging from 0.81 to 0.83 (Gould et al., 2016). In the present study, the Cronbach’s alpha coefficient for the CES-D was 0.767, indicating acceptable internal consistency. A cutoff score of 4 or higher was used to indicate elevated depressive symptoms (Gould et al., 2016).

Anxiety symptoms were assessed using the 5-item version of the Beck Anxiety Inventory (BAI) included in the HRS psychosocial questionnaire (Guide to Content of the HRS Psychosocial Leave-Behind Participant Lifestyle Questionnaires: 2004 and 2006, 2008). The items asked whether the participant worried about the worst happening, felt nervous, felt their hands trembling, feared dying, or felt faint. Based on their experiences during the past week, participants rated the frequency of each symptom on a 4-point scale ranging from “never” to “most of the time” (Beck et al., 2013), scored from 1 to 4. Total scores ranged from 5 to 20, with higher scores indicating more severe anxiety symptoms. In this study, a total score of 12 or higher was used to indicate the presence of anxiety symptoms (Gould et al., 2015). The Cronbach’s alpha c`oefficient for the BAI in the present sample was 0.820, indicating good internal consistency.

#### 2.2.3 Covariates and potential mediators

Based on previous literature and theoretical relevance, sex, language, age, race, education, marital status, total household income, life satisfaction, health status, chronic conditions, eyesight, BMI category, and sleep disorder were included as covariates in the multivariable models. Anxiety, health status, and eyesight were also included in the mediation analysis of the association between depression and cognitive function.

### 2.3 Statistical analysis

Data were cleaned, coded, and screened for invalid cases using Excel. All statistical analyses were conducted in R. Categorical variables are presented as frequencies and percentages, and group differences were assessed using the chi-square test. Cognitive function status (normal cognition, CIND, and dementia) was treated as the outcome variable in the multivariable analysis, and associated factors were examined using ordered logistic regression. The multivariable model was fitted using the Enter method, with all prespecified covariates entered simultaneously; no stepwise variable selection procedure was performed. Ordered logistic regression was conducted using the MASS package. Mediation analyses were conducted using the lavaan package to examine the mediating roles of anxiety, health status, and eyesight in the association between depression and cognitive function. In the mediation models, cognitive function was treated as a continuous outcome in order to preserve score variation and facilitate estimation of indirect effects, and bias-corrected bootstrap confidence intervals were estimated using 5,000 resamples.

## 3 Results

### 3.1 Participant characteristics

Table 1 summarizes the characteristics of the 3,434 participants. The mean age was 68.7 years (SD = 10.2), and 58.6% were female. Most interviews were conducted in English (92.9%). The prevalence of depression and anxiety was 12.3 and 12.8%, respectively. Based on cognitive function classification, 83.8% of participants were cognitively normal, 13.7% had CIND, and 2.4% had dementia, indicating that 16.2% of the sample had cognitive impairment overall.

**Table 1:** Demographic characteristics of the elderly.

Variable	Group	*N*	Proportion (%)
Sex	Male	1,422	41.4%
	Female	2012	58.6%
Language	English	3,190	92.9%
	Spanish	244	7.1%
Age	50–59	596	17.4%
	60–69	1,267	36.9%
	70–79	975	28.4%
	80-	596	17.4%
Race	White/Caucasian	2,432	70.8%
	Black/African American	646	18.8%
	Other	356	10.4%
Education	Lt HS	347	10.1%
	HS/GED	1,690	49.2%
	AA	260	7.6%
	BA+	1,137	33.1%
Marital status	Married	2001	58.3%
	Separated/Divorced	617	18.0%
	Widowed	557	16.2%
	Never married	259	7.5%
Total household income	Lowest income	865	25.2%
	Lower-middle income	854	24.9%
	Upper-middle income	856	24.9%
	Highest income	859	25%
Life satisfaction	Satisfied	3,323	96.8%
	Unsatisfied	111	3.2%
Health status	Good	2,667	77.7%
	Normal	638	18.6%
	Poor	129	3.8%
High blood pressure	No	1,329	38.7%
	Yes	2,105	61.3%
Diabetes	No	2,468	71.9%
	Yes	966	28.1%
Lung disease	No	3,064	89.2%
	Yes	370	10.8%
Heart condition	No	2,566	74.7%
	Yes	868	25.3%
Stroke	No	3,227	94.0%
	Yes	207	6.0%
Arthritis	No	1,365	39.7%
	Yes	2069	60.3%
Eyesight	Good	2,708	78.9%
	Fair	562	16.4%
	Poor	164	4.8%
BMI category	Non-overweight	763	22.2%
	Overweight	1,171	34.1%
	Obesity	1,500	43.7%
Sleep disorder	No	2,716	79.1%
	Yes	718	20.9%
Anxiety	No	2,994	87.2%
	Yes	440	12.8%
Cognitive Function	Normal	2,878	83.8%
	CIND	472	13.7%
	Dementia	84	2.4%

### 3.2 Factors associated with cognitive function status: univariate and multivariate analyses

The results of the univariate analyses are presented in Table 2. Cognitive impairment was significantly associated with language, age, race, educational level, marital status, total household income, life satisfaction, health status, high blood pressure, diabetes, lung disease, heart condition, stroke, arthritis, eyesight, BMI category, anxiety, and depression (all *p* < 0.05). No significant association was found for sex or sleep disorder (both *p* > 0.05).

**Table 2:** Influencing factors of cognitive function in the elderly.

Variable	Group	Cognitive function			Total	*χ* ^2^	*p*
		Normal (%)	CIND (%)	Dementia (%)			
Sex	Male	1,200 (84.4)	196 (13.8)	26 (1.8)	1,422	3.886	0.143
	Female	1,678 (83.4)	276 (13.7)	58 (2.9)	2012		
Language	English	2,707 (84.9)	410 (12.9)	73 (2.3)	3,190	36.474	<0.001
	Spanish	171 (70.1)	62 (25.4)	11 (4.5)	244		
Age	50–59	534 (89.6)	55 (9.2)	7 (1.2)	596	92.193	<0.001
	60–69	1,115 (88.0)	132 (10.4)	20 (1.6)	1,267		
	70–79	795 (81.5)	154 (15.8)	26 (2.7)	975		
	80-	434 (72.8)	131 (22.0)	31 (5.2)	596		
Race	White/Caucasian	2,131 (87.6)	273 (11.2)	28 (1.2)	2,432	115.54	<0.001
	Black/African American	472 (73.1)	133 (20.6)	41 (6.3)	646		
	Other	275 (77.2)	66 (18.5)	15 (4.2)	356		
Education	Lt HS	185 (53.3)	121 (34.9)	41 (11.8)	347	399.534	<0.001
	HS/GED	1,379 (81.6)	276 (16.3)	35 (2.1)	1,690		
	AA	233 (89.6)	23 (8.8)	4 (1.5)	260		
	BA+	1,081 (95.1)	52 (4.6)	4 (0.4)	1,137		
Marital status	Married	1726 (86.3)	240 (12.0)	35 (1.7)	2001	31.397	<0.001
	Separated/Divorced	508 (82.3)	92 (14.9)	17 (2.8)	617		
	Widowed	431 (77.4)	101 (18.1)	25 (4.5)	557		
	Never married	213 (82.2)	39 (15.1)	7 (2.7)	259		
Total household income	Lowest income	595 (68.8)	217 (25.1)	53 (6.1)	865	268.35	<0.001
	Lower-middle income	700 (82.0)	140 (16.4)	14 (1.6)	854		
	Upper-middle income	758 (88.6)	84 (9.8)	14 (1.6)	856		
	Highest income	825 (96.0)	31 (3.6)	3 (0.3)	859		
Life satisfaction	Satisfied	2,797 (84.2)	446 (13.4)	80 (2.4)	3,323	10.053	0.007
	Unsatisfied	81 (73.0)	26 (23.4)	4 (3.6)	111		
Health status	Good	2,330 (87.4)	294 (11.0)	43 (1.6)	2,667	123.523	<0.001
	Normal	466 (73.0)	140 (21.9)	32 (5.0)	638		
	Poor	82 (63.6)	38 (29.5)	9 (7.0)	129		
High blood pressure	No	1,193 (89.8)	118 (8.9)	18 (1.4)	1,329	57.096	<0.001
	Yes	1,685 (80.0)	354 (16.8)	66 (3.1)	2,105		
Diabetes	No	2,118 (85.8)	296 (12.0)	54 (2.2)	2,468	26.196	<0.001
	Yes	760 (78.7)	176 (18.2)	30 (3.1)	966		
Lung disease	No	2,597 (84.8)	398 (13.0)	69 (2.3)	3,064	19.246	<0.001
	Yes	281 (75.9)	74 (20.0)	15 (4.1)	370		
Heart condition	No	2,179 (84.9)	332 (12.9)	55 (2.1)	2,566	10.097	0.006
	Yes	699 (80.5)	140 (16.1)	29 (3.3)	868		
Stroke	No	2,729 (84.6)	425 (13.2)	73 (2.3)	3,227	23.951	<0.001
	Yes	149 (72.0)	47 (22.7)	11 (5.3)	207		
Arthritis	No	1,185 (86.8)	156 (11.4)	24 (1.8)	1,365	15.666	<0.001
	Yes	1,693 (81.8)	316 (15.3)	60 (2.9)	2069		
Eyesight	Good	2,347 (86.7)	314 (11.6)	47 (1.7)	2,708	83.741	<0.001
	Fair	414 (73.7)	118 (21.0)	30 (5.3)	562		
	Poor	117 (71.3)	40 (24.4)	7 (4.3)	164		
BMI category	Non-overweight	612 (80.2)	124 (16.3)	27 (3.5)	763	12.493	0.014
	Overweight	992 (84.7)	149 (12.7)	30 (2.6)	1,171		
	Obesity	1,274 (84.9)	199 (13.3)	27 (1.8)	1,500		
Sleep disorder	No	2,294 (84.5)	360 (13.3)	62 (2.3)	2,716	4.349	0.114
	Yes	584 (81.3%)	112 (15.6)	22 (3.1)	718		
Anxiety	No	2,561 (85.5%)	377 (12.6)	56 (1.9)	2,994	62.597	<0.001
	Yes	317 (72%)	95 (21.6)	28 (6.4)	440		
Depression	No	2,593 (86.1%)	361 (12.0)	59 (2.0)	3,013	94.391	<0.001
	Yes	285 (67.7%)	111 (26.4)	25 (5.9)	421		

**Figure 1:**
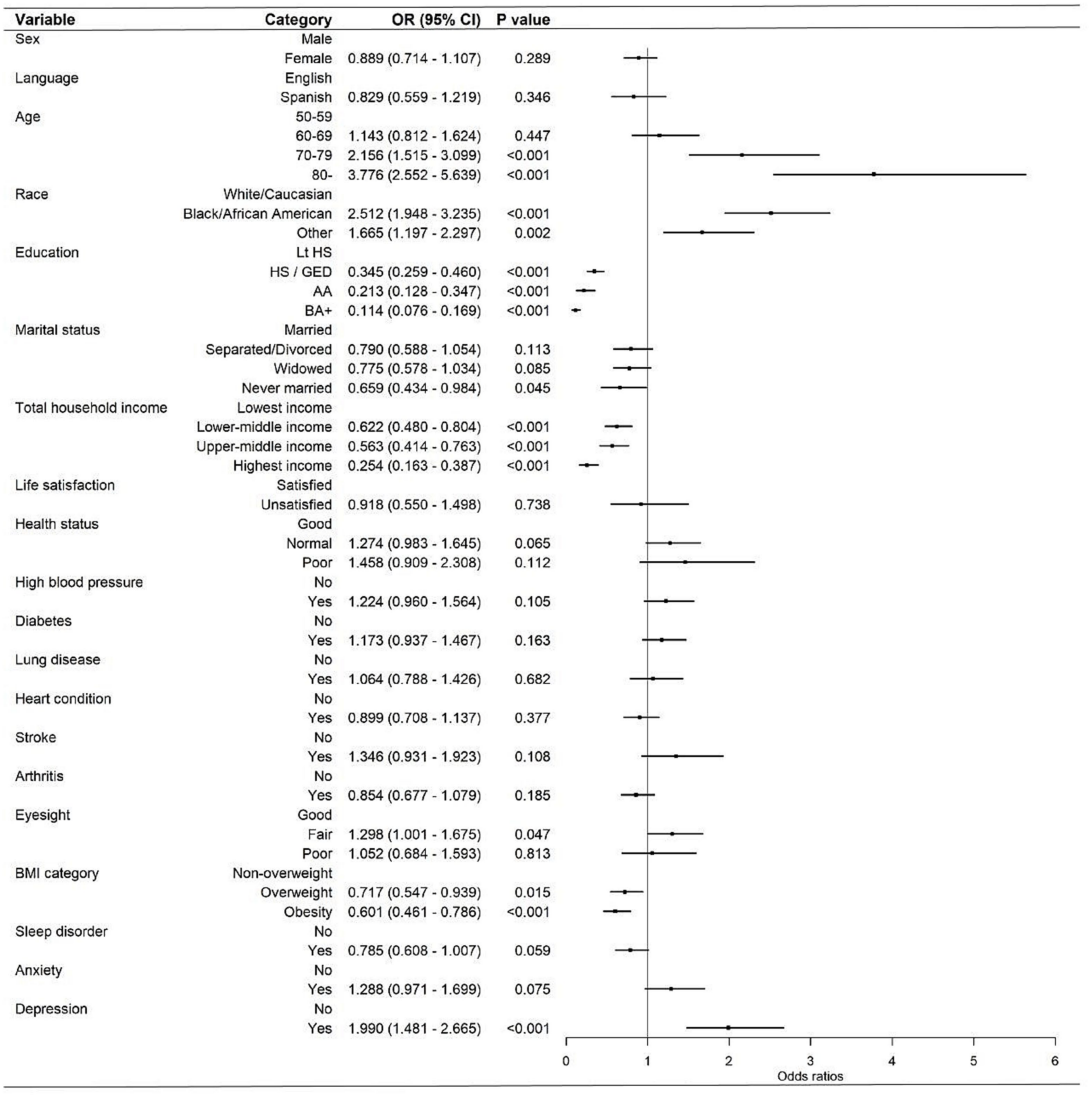
Forest plot of multivariate binary logistic regression analysis.

Table 3 presents the results of the fully adjusted ordered logistic regression model. After adjustment for potential confounders, age, race, education, marital status, total household income, eyesight, BMI category, and depression were significantly associated with cognitive function status in the fully adjusted model. Compared with participants aged 50–59 years, those aged 70–79 years (OR = 2.156, 95% CI: 1.515–3.099) and 80 years or older (OR = 3.776, 95% CI: 2.552–5.639) had significantly higher odds of being in a worse cognitive function category. Compared with White/Caucasian participants, Black/African American participants (OR = 2.512, 95% CI: 1.948–3.235) and participants of other races (OR = 1.665, 95% CI: 1.197–2.297) were more likely to have worse cognitive function status. Higher educational attainment was associated with lower odds of worse cognitive function status, including HS/GED (OR = 0.345, 95% CI: 0.259–0.460), AA (OR = 0.213, 95% CI: 0.128–0.347), and BA+ (OR = 0.114, 95% CI: 0.076–0.169), compared with less than high school education. Never-married participants had lower odds of worse cognitive function status than married participants (OR = 0.659, 95% CI: 0.434–0.984). In addition, compared with the lowest-income group, those in the lower-middle-income (OR = 0.622, 95% CI: 0.480–0.804), upper-middle-income (OR = 0.563, 95% CI: 0.414–0.763), and highest-income groups (OR = 0.254, 95% CI: 0.163–0.387) had significantly lower odds of worse cognitive function status. Fair eyesight was associated with higher odds of worse cognitive function status compared with good eyesight (OR = 1.298, 95% CI: 1.001–1.675). Being overweight (OR = 0.717, 95% CI: 0.547–0.939) or obese (OR = 0.601, 95% CI: 0.461–0.786) was associated with lower odds of worse cognitive function status compared with being non-overweight. Depression was also significantly associated with cognitive function status, with depressed participants showing higher odds of worse cognitive function status (OR = 1.990, 95% CI: 1.481–2.665). In contrast, sex, language, life satisfaction, health status, high blood pressure, diabetes, lung disease, heart condition, stroke, arthritis, sleep disorder, and anxiety were not significantly associated with cognitive function status in the fully adjusted model (all *p* > 0.05). The corresponding forest plot is shown in Figure 1.

**Table 3:** Multivariate logistic regression analysis of cognitive function influencing factors.

Variable	Group	*β*	S. E.	Wald statistic	*p*	OR	95%CI	
							Lower limit	Upper limit
Sex	Male	–	–	–	–	–	–	–
	Female	−0.118	0.112	1.110	0.289	0.889	0.714	1.107
Language	English	–	–	–	–	–	–	–
	Spanish	−0.187	0.199	0.890	0.346	0.829	0.559	1.219
Age	50–59	–	–	–	–	–	–	–
	60–69	0.134	0.176	0.580	0.447	1.143	0.812	1.624
	70–79	0.768	0.182	17.760	<0.001	2.156	1.515	3.099
	80	1.329	0.202	43.230	<0.001	3.776	2.552	5.639
Race	White/Caucasian	–	–	–	–	–	–	–
	Black/African American	0.921	0.129	50.740	<0.001	2.512	1.948	3.235
	Other	0.51	0.166	9.420	0.002	1.665	1.197	2.297
Education	Lt HS	–	–	–	–	–	–	–
	HS/GED	−1.063	0.147	52.620	<0.001	0.345	0.259	0.46
	AA	−1.544	0.254	37.010	<0.001	0.213	0.128	0.347
	BA+	−2.171	0.202	115.510	<0.001	0.114	0.076	0.169
Marital status	Married	–	–	–	–	–	–	–
	Separated/Divorced	−0.236	0.149	2.510	0.113	0.79	0.588	1.054
	Widowed	−0.255	0.148	2.970	0.085	0.775	0.578	1.034
	Never married	−0.417	0.208	4.010	0.045	0.659	0.434	0.984
Total household income	Lowest income	–	–	–	–	–	–	–
	Lower-middle income	−0.475	0.132	13.020	<0.001	0.622	0.48	0.804
	Upper-middle income	−0.574	0.156	13.570	<0.001	0.563	0.414	0.763
	Highest income	−1.37	0.219	39.070	<0.001	0.254	0.163	0.387
Life satisfaction	Satisfied	–	–	–	–	–	–	–
	Unsatisfied	−0.085	0.255	0.110	0.738	0.918	0.55	1.498
Health status	Good	–	–	–	–	–	–	–
	Normal	0.242	0.131	3.400	0.065	1.274	0.983	1.645
	Poor	0.377	0.237	2.520	0.112	1.458	0.909	2.308
High blood pressure	No	–	–	–	–	–	–	–
	Yes	0.202	0.124	2.630	0.105	1.224	0.96	1.564
Diabetes	No	–	–	–	–	–	–	–
	Yes	0.16	0.114	1.950	0.163	1.173	0.937	1.467
Lung disease	No	–	–	–	–	–	–	–
	Yes	0.062	0.151	0.170	0.682	1.064	0.788	1.426
Heart condition	No	–	–	–	–	–	–	–
	Yes	−0.107	0.121	0.780	0.377	0.899	0.708	1.137
Stroke	No	–	–	–	–	–	–	–
	Yes	0.297	0.185	2.590	0.108	1.346	0.931	1.923
Arthritis	No	–	–	–	–	–	–	–
	Yes	−0.157	0.119	1.760	0.185	0.854	0.677	1.079
Eyesight	Good	–	–	–	–	–	–	–
	Fair	0.261	0.131	3.950	0.047	1.298	1.001	1.675
	Poor	0.051	0.216	0.060	0.813	1.052	0.684	1.593
BMI category	Non-overweight	–	–	–	–	–	–	–
	Overweight	−0.333	0.138	5.860	0.015	0.717	0.547	0.939
	Obesity	−0.509	0.136	13.980	<0.001	0.601	0.461	0.786
Sleep disorder	No	–	–	–	–	–	–	–
	Yes	−0.242	0.129	3.560	0.059	0.785	0.608	1.007
Anxiety	No	–	-	–	–	–	–	–
	Yes	0.253	0.143	3.160	0.075	1.288	0.971	1.699
Depression	No	–	–	–	–	–	–	–
	Yes	0.688	0.150	21.060	<0.001	1.99	1.481	2.665

### 3.3 Mediation analysis results

To explore the underlying mechanism linking depression and cognitive function, anxiety, health status, and eyesight were separately introduced as mediators and tested using the lavaan package in R. Gender, language, age, race, educational level, marital status, total household income, hypertension, diabetes, lung disease, heart disease, stroke, arthritis, sleep disorder, and BMI category were included as covariates. Cognitive function was treated as a continuous outcome variable ranging from 0 to 27, with higher scores indicating better cognitive function. A bias-corrected bootstrap method with 5,000 resamples was used to estimate the 95% confidence intervals of the indirect effects.

**Figure 2:**
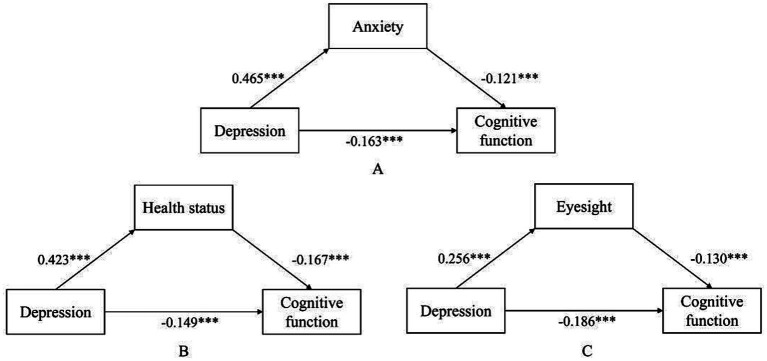
Path coefficients of the mediation models of anxiety, health status, and eyesight in the relationship between depression and cognitive function. ***p < 0.001,**p < 0.01, *p < 0.05.

The regression results showed that depression significantly and negatively predicted cognitive function (*β* = −0.220, *p* < 0.001), indicating that higher levels of depression were associated with poorer cognitive function. After adding the mediators, the predictive effect of depression on cognitive function remained significant in all three models. Specifically, in the anxiety mediation model, the direct effect of depression on cognitive function was *β* = −0.163 (*p* < 0.001), and anxiety was significantly associated with cognitive function (*β* = −0.121, *p* < 0.001). In the health status mediation model, the direct effect of depression on cognitive function was *β* = −0.149 (*p* < 0.001), and health status was significantly associated with cognitive function (*β* = −0.167, *p* < 0.001). In the eyesight mediation model, the direct effect of depression on cognitive function was *β* = −0.186 (*p* < 0.001), and eyesight was significantly associated with cognitive function (*β* = −0.130, *p* < 0.001). The regression results are presented in Table 4, and the path coefficients are shown in Figure 2.

**Table 4:** Regression analysis of the mediating roles of anxiety, health status, and eyesight in the relationship between depression and cognitive function.

Model	Effect	Effect value	SE	LLCI	ULCI	Effect size
Model 1: anxiety	Total effect	−0.220	0.017	−0.253	−0.186	–
	Direct effect	−0.163	0.020	−0.202	−0.126	74.34%
	Indirect effect	−0.056	0.009	−0.075	−0.038	25.66%
Model 2: health status	Total effect	−0.220	0.017	−0.253	−0.186	–
	Direct effect	−0.149	0.019	−0.186	−0.112	67.81%
	Indirect effect	−0.071	0.008	−0.088	−0.055	32.19%
Model 3: eyesight	Total effect	−0.220	0.017	−0.253	−0.186	–
	Direct effect	−0.186	0.018	−0.220	−0.152	84.81%
	Indirect effect	−0.033	0.005	−0.044	−0.024	15.19%

The mediation analyses further indicated that anxiety, health status, and eyesight all significantly mediated the association between depression and cognitive function. The indirect effect of anxiety was −0.056 (95% CI: −0.075, −0.038), accounting for 25.66% of the total effect. The indirect effect of health status was −0.071 (95% CI: −0.088, −0.055), accounting for 32.19% of the total effect. The indirect effect of eyesight was −0.033 (95% CI: −0.044, −0.024), accounting for 15.19% of the total effect. Among the three mediators, health status showed the largest mediating effect, followed by anxiety and eyesight. The decomposition of the mediation effects is presented in Table 5.

**Table 5:** Decomposition of the total, direct, and indirect effects of anxiety, health status, and eyesight in the relationship between depression and cognitive function.

Model	Regression equations		Overall fit index			Regression coefficient *β*	*t*-value
	Outcome variable	Predictor variable	*R*	*R* ^2^	*F*-value		
Total effect model	Cognitive function	Depression	0.220	0.048	173.870	−0.220	13.186***
Anxiety mediation	Anxiety	Depression	0.465	0.216	944.991	0.465	30.741***
	Cognitive function	Anxiety	0.244	0.060	109.013	−0.121	6.487***
		Depression				−0.163	8.732***
Health status mediation	Health status	Depression	0.423	0.179	749.706	0.423	27.381***
	Cognitive function	Health status	0.267	0.071	131.277	−0.167	9.190***
		Depression				−0.149	8.199***
Eyesight mediation	Eyesight	Depression	0.256	0.065	240.065	0.256	15.494***
	Cognitive function	Eyesight	0.253	0.064	117.545	−0.130	7.636***
		Depression				−0.186	10.901***

*** *p* < 0.001.

## 4 Discussion

In the present study, the distribution of cognitive function status among adults aged 50 years and older was as follows: 83.8% were cognitively normal, 13.7% had cognitive impairment no dementia (CIND), and 2.4% had dementia. This pattern was similar to that reported in some previous studies but differed from others (Liao et al., 2022; Haque et al., 2025; Shi et al., 2025), suggesting that the distribution of cognitive function status may be influenced by methodological differences, cultural background, and population characteristics.

Our findings further indicated that cognitive function status in middle-aged and older adults was associated with multiple demographic and socioeconomic factors. Among these, age remained an important factor related to cognitive decline. Compared with participants aged 50–59 years, those aged 70–79 years and those aged 80 years and older had 2.156-fold and 3.776-fold higher odds of being in a worse cognitive function category, respectively, which is consistent with previous findings (Liu et al., 2024; Tu et al., 2024). Relevant studies have shown that, during the aging process, structural and functional changes occur in the brain, such as brain atrophy, increased neuronal vulnerability, and decline in the function of memory-related brain regions, all of which may contribute to cognitive decline (Xia et al., 2018).

In addition to age, this study also found that race, educational level, marital status, and total household income were statistically associated with cognitive function status, which is broadly consistent with previous findings (Meng and D’Arcy, 2012; Sommerlad et al., 2018; Liao et al., 2022; Wang et al., 2023; Shi et al., 2025). Higher educational attainment was associated with a lower likelihood of being in a worse cognitive function category, which is consistent with the cognitive reserve hypothesis and suggests that education may play a protective role in maintaining cognitive function (Meng and D’Arcy, 2012). Higher total household income was also associated with better cognitive function status, which may be related to the advantages of individuals with higher socioeconomic status in access to healthcare resources, nutritional conditions, and health management (Wang et al., 2023). At the same time, differences were observed across racial groups, which may be related to social environment, health inequalities, and disparities in access to relevant resources (Beydoun et al., 2022).

Regarding health-related factors, this study found a statistically significant association between eyesight and cognitive function status. Compared with participants with good eyesight, those with fair eyesight were more likely to be in a worse cognitive function category, which is consistent with the findings (Li et al., 2017). This may be related to age-related visual decline, damage to the suprachiasmatic nucleus, and loss of cholinergic neurons in the basal forebrain (Li et al., 2017). In addition, compared with non-overweight participants, overweight and obese participants were less likely to be in a worse cognitive function category. The relationship between BMI and cognitive function may be complex and may be influenced by factors such as age structure, nutritional status, chronic disease-related wasting, and the choice of reference group; therefore, this finding should be interpreted with caution.

In addition, health status, hypertension, diabetes, and stroke were associated with cognitive function status in the univariate analysis. Previous studies have suggested that chronic non-communicable diseases such as hypertension, diabetes, and stroke may be related to cognitive decline (Ye and Wang, 2025; Shi et al., 2025). Chronic diseases may reduce quality of life and thereby affect cognitive function, and older adults often require long-term use of multiple medications, the side effects of which may lead to memory decline and inattention, further affecting cognitive function (Ye and Wang, 2025). However, these variables were not statistically significant in the ordered logistic regression analysis in the present study, suggesting that their relationships with cognitive function may be influenced by the combined effects of other demographic and socioeconomic factors.

This study also showed a significant association between depression and worse cognitive function status. The proportion of cognitive dysfunction was markedly higher among older adults with depression than among those without depression. In the ordered logistic regression analysis, depressed participants had 1.990 times the odds of being in a worse cognitive function category compared with non-depressed participants, which is consistent with the findings (Sun et al., 2024). Depression may be associated with cognitive decline through pathways such as reduced social participation, decreased cognitive activity, and disturbances in sleep and appetite (Sun et al., 2024; Kang and Cao, 2024). In this study, anxiety was associated with cognitive function status in the univariate analysis, but it was not statistically significant in the ordered logistic regression analysis, suggesting that the relationship between anxiety and cognitive function may be more complex. However, the subsequent mediation analysis showed that anxiety had a significant indirect effect in the association between depression and cognitive function, indicating that the potential role of anxiety still deserves attention (Santabárbara et al., 2019).

The mediation analysis further showed that anxiety, health status, and eyesight all served as partial mediators in the association between depression and cognitive function in middle-aged and older adults, accounting for 25.66, 32.19, and 15.19% of the total effect, respectively. After anxiety, health status, and eyesight were included in the model, the direct effect of depression on cognitive function remained significant, but its magnitude was attenuated, suggesting that these variables may partly account for the association between depression and cognitive function. Among them, health status accounted for the largest indirect effect. This may be because depression can influence individuals’ health perceptions, self-care behaviors, and lifestyles, while poorer health status is in turn associated with cognitive decline (Montlahuc et al., 2011; Peleg and Nudelman, 2021). The indirect effect of anxiety suggests that psychological factors may also play a role, possibly because excessive worry, tension, vigilance, and rumination in anxious states consume cognitive resources (Santabárbara et al., 2019). The significant indirect effect of eyesight suggests that sensory function may also be involved in the association between depression and cognitive function, possibly because declining visual function reduces sensory input, lowers social participation, and increases cognitive processing burden (Nagarajan et al., 2022; Virgili et al., 2022).

### 4.1 Limitations and strengths

This study still has several limitations. First, because cross-sectional data were used, the temporal order among the variables could not be determined. Therefore, the findings, especially those from the mediation analysis, should be interpreted as statistical associations and potential pathways rather than as strict causal relationships. Second, several measures were based on self-report, and cognitive function was assessed by telephone, which may have introduced recall bias, reporting bias, and measurement error. Although the 5-item BAI included in the HRS is appropriate for large-scale population surveys, it may not capture anxiety symptoms as comprehensively as the full instrument. Third, although multiple demographic and health-related variables were included, residual confounding may still exist, and excluding participants with missing data on key variables may also have introduced selection bias. Finally, this study was based on HRS data and mainly reflects the characteristics of older adults in the United States; therefore, the generalizability of the findings to other cultural and social contexts requires further verification. Fifth, the Cronbach’s alpha coefficient of the TICS-27 scale used to assess cognitive function was 0.660 in this study, which is below the generally accepted standard of 0.70. Since TICS-27 was used to classify participants into normal cognition, CIND, and probable dementia groups—the key outcome variable in this study—this relatively low reliability may affect the accuracy of cognitive classification and represents an important methodological limitation.

This study also has several strengths. First, it was based on the large 2022 HRS dataset, with a relatively large sample size and good population representativeness, providing a reliable basis for analyzing factors related to cognitive function status in middle-aged and older adults. Second, unlike previous studies that simply dichotomized cognitive outcomes into normal versus abnormal, this study classified cognitive outcomes into normal, CIND, and dementia, and applied ordered logistic regression to analyze factors associated with cognitive function status, thereby better capturing the gradient differences in cognitive status. Third, this study not only focused on the association between depression and cognitive function but also further examined the potential roles of anxiety, health status, and eyesight, thereby expanding understanding of factors related to cognitive function in older adults from psychological, general health, and sensory perspectives. Finally, CES-D, BAI, and TICS-27 were used to measure depression, anxiety, and cognitive function, respectively, and these instruments showed good reliability, providing a solid measurement basis for the study findings.

### 4.2 Conclusion and implications

In summary, this study, based on data from the 2022 Health and Retirement Study, found that age, race, educational level, marital status, total household income, eyesight, BMI category, and depression were associated with cognitive function status among middle-aged and older adults. Mediation analysis further suggested that anxiety, health status, and eyesight may play partial roles in the association between depression and cognitive function. These findings suggest that psychological status, general health, and sensory function should all be considered in understanding cognitive function in middle-aged and older adults, and may help inform the early identification and comprehensive intervention of cognitive decline.

## Author’s note

2023 Autonomous Region-level College Student Innovation and Entrepreneurship Project: “Retaining People with ‘Emotion’: A Study on the Emotional Labor of Rural Family Doctors”. 2025 University-level College Student Innovation and Entrepreneurship Project: “The Way of Family Harmony: Decoding the Enigma of Emotional Cold Violence”.

## Data Availability

The datasets analyzed for this study can be found on the Health and Retirement Study website: https://hrs.isr.umich.edu/ (Accessed June 1, 2025).

## References

[ref1] Beck A. Steer R. Beck A. F. Y. Steer R. (2013). Manual for the Beck Anxiety Inventory. San Antonio, TX, USA: Psychological Corporation. Available online at: https://xueshu.baidu.com/usercenter/paper/show?paperid=1v0x0ve0f40502r0g06n04p0ww646020 (Accessed April 18, 2026).

[ref2] Beekman A. T. de Beurs E. van Balkom A. J. Deeg D. J. van Dyck R. van Tilburg W. (2000). Anxiety and depression in later life: co-occurrence and communality of risk factors. Am. J. Psychiatry 157, 89–95. doi: 10.1176/ajp.157.1.89, 10618018

[ref3] Beydoun M. A. Beydoun H. A. Banerjee S. Weiss J. Evans M. K. Zonderman A. B. (2022). Pathways explaining racial/ethnic and socio-economic disparities in incident all-cause dementia among older US adults across income groups. Transl. Psychiatry 12:478. doi: 10.1038/s41398-022-02243-y, 36379922 PMC9666623

[ref4] Chinese Expert Consensus Group on Prevention and Treatment of Cognitive Dysfunction (2006). Chinese expert consensus on prevention and treatment of cognitive dysfunction. Chin. J. Inter. Med. 45, 171–173. doi: 10.3760/j:issn:0254-9026.2006.07.001

[ref5] Crimmins E. M. Kim J. K. Langa K. M. Weir D. R. (2011). Assessment of cognition using surveys and neuropsychological assessment: the health and retirement study and the aging, demographics, and memory study. J. Gerontol. B Psychol. Sci. Soc. Sci. 66, i162–i171. doi: 10.1093/geronb/gbr048, 21743047 PMC3165454

[ref6] Ghisletta P. Rabbitt P. Lunn M. Lindenberger U. (2012). Two thirds of the age-based changes in fluid and crystallized intelligence, perceptual speed, and memory in adulthood are shared. Intelligence 40, 260–268. doi: 10.1016/j.intell.2012.02.008

[ref7] Gould C. E. O’Hara R. Goldstein M. K. Beaudreau S. A. (2016). Multimorbidity is associated with anxiety in older adults in the health and retirement study. Int. J. Geriatr. Psychiatry 31, 1105–1115. doi: 10.1002/gps.4532, 27441851 PMC5312684

[ref8] Gould C. E. Rideaux T. Spira A. P. Beaudreau S. A. (2015). Depression and anxiety symptoms in male veterans and non-veterans: the health and retirement study. Int. J. Geriatr. Psychiatry 30, 623–630. doi: 10.1002/gps.4193, 25145943 PMC4336840

[ref9] Guide to Content of the HRS Psychosocial Leave-Behind Participant Lifestyle Questionnaires: 2004 and 2006 (2008). Kipdf.com. Available online at: https://kipdf.com/guide-to-content-of-the-hrs-psychosocial-leave-behind-participant-lifestyle-ques_5ad997867f8b9a70308b45b6.html (Accessed April 18, 2026).

[ref10] Hanes D. W. Clouston S. A. P. (2023). Cognitive aging in same- and different-sex relationships: comparing age of diagnosis and rate of cognitive decline in the health and retirement study. Gerontology 69, 356–369. doi: 10.1159/000526922, 36509083 PMC9991936

[ref11] Haque R. Alam K. Gow J. Neville C. Keramat S. A. (2025). Cognitive impairment and self-reported health outcomes among older adults: longitudinal evidence from Australia. Acta Psychol. 253:104770. doi: 10.1016/j.actpsy.2025.104770, 39892103

[ref12] Ismail Z. Elbayoumi H. Fischer C. E. Hogan D. B. Millikin C. P. Schweizer T. . (2017). Prevalence of depression in patients with mild cognitive impairment: a systematic review and Meta-analysis. JAMA Psychiatry 74, 58–67. doi: 10.1001/jamapsychiatry.2016.3162 27893026

[ref13] Kang Z. X. Cao Y. (2024). Relationship between depression score and cognitive ability in elderly patients with depression. Heilongjiang Science 15, 144–146. doi: 10.1016/j.ebiom.2024.105255

[ref14] Karlamangla A. S. Miller-Martinez D. Aneshensel C. S. Seeman T. E. Wight R. G. Chodosh J. (2009). Trajectories of cognitive function in late life in the United States: demographic and socioeconomic predictors. Am. J. Epidemiol. 170, 331–342. doi: 10.1093/aje/kwp154, 19605514 PMC2727175

[ref15] Larner A. (2008). “Cognitive function, neuropsychological evaluation, and syndromes of cognitive impairment,” in Neuropsychological Neurology: The Neurocognitive Impairments of Neurological Disorders. J. ed (Cambridge: Cambridge University Press), 6–38. doi: 10.1017/CBO9780511545009.002

[ref16] Li M. Q. Huang H. H. Mou X. Jiang G. X. Chen Q. H. (2017). Prevalence and influencing factors of cognitive dysfunction among the elderly aged 70 years and above in Jianghan oilfield. Chin. J. Geriatric Heart Brain Vessel Diseases 19, 1137–1141. doi: 10.3389/fpsyg.2017.01334

[ref17] Liang M. Zhang M. Q. Mei X. Xu Y. Q. (2025). Correlation between BDNF gene polymorphism and thyroid hormone levels in elderly patients with depressive symptoms. J. Environ. Health 42, 95–101. doi: 10.16241/j.cnki.1001-5914.2025.02.001

[ref18] Liao T. T. Lin L. F. Xu H. F. Meng R. L. Zheng X. Y. Peng D. D. . (2022). Prevalence and influencing factors of cognitive impairment among the elderly in Guangdong Province. Modern Prevent. Med. 49, 107–109. doi: 10.1016/j.jad.2022.08.009

[ref19] Liu Z. J. Wang L. L. Wang C. X. (2024). Current status and influencing factors of cognitive dysfunction among the elderly in Chengyang District, Qingdao. J. Qingdao Univ. 60, 767–770. doi: 10.3969/j.issn.1004-583X.2024.04.004

[ref20] Livingston G. Huntley J. Sommerlad A. Ames D. Ballard C. Banerjee S. . (2020). Dementia prevention, intervention, and care: 2020 report of the lancet commission. Lancet 396, 413–446. doi: 10.1016/S0140-6736(20)30367-6, 32738937 PMC7392084

[ref21] Lu S. M. Feng X. Y. Tang W. Ma M. N. Ni C. P. Liu J. L. . (2020). Effects of computerized cognitive training on depression and anxiety in cognitively healthy older adults: a meta-analysis. Chin. Nurs. Manag. 20, 881–887. doi: 10.3969/j.issn.1672-1756.2020.06.017

[ref22] Meng X. D’Arcy C. (2012). Education and dementia in the context of the cognitive reserve hypothesis: a systematic review with meta-analyses and qualitative analyses. PLoS One 7:e38268. doi: 10.1371/journal.pone.0038268 22675535 PMC3366926

[ref23] Mezuk B. Kershaw K. N. Hudson D. Lim K. A. Ratliff S. (2011). Job strain, workplace discrimination, and hypertension among older workers: the health and retirement study. Race Soc. Probl. 3, 38–50. doi: 10.1007/s12552-011-9041-7, 22096475 PMC3215400

[ref24] Mitchell O. S. Clark R. Lusardi A. (2022). Income trajectories in later life: longitudinal evidence from the health and retirement study. J. Econ. Ageing 22:100371. doi: 10.1016/j.jeoa.2022.100371, 36156898 PMC9502039

[ref25] Montlahuc C. Soumaré A. Dufouil C. Berr C. Dartigues J.-F. Poncet M. . (2011). Self-rated health and risk of incident dementia: a community-based elderly cohort, the 3C study. Neurology 77, 1457–1464. doi: 10.1212/WNL.0b013e31823303e1 21975209

[ref26] Nagarajan N. Assi L. Varadaraj V. Motaghi M. Sun Y. Couser E. . (2022). Vision impairment and cognitive decline among older adults: a systematic review. BMJ Open 12:e047929. doi: 10.1136/bmjopen-2020-047929, 34992100 PMC8739068

[ref27] Natale G. Clouston S. A. P. Smith D. M. (2022). Elevated C-reactive protein in Alzheimer’s disease without depression in older adults: findings from the health and retirement study. J. Gerontol. A Biol. Sci. Med. Sci. 77, 673–682. doi: 10.1093/gerona/glab282 34671810 PMC8974321

[ref28] National Bureau of Statistics of China (2024). China Statistical Yearbook 2024. Beijing, China: China Statistics Press. Available online at: https://www.stats.gov.cn/sj/ndsj/2024/indexch.htm (Accessed April 18, 2026).

[ref29] National Health Commission of the People’s Republic of China (2022). Notice on Issuing the “14th Five-Year Plan” for Healthy Aging. Gazette of the State Council of the People’s Republic of China. Beijing, China: National Health Commission of the People’s Republic of China. Available online at: https://www.gov.cn/gongbao/content/2022/content_5692863.htm (Accessed April 18, 2026).

[ref30] Peleg S. Nudelman G. (2021). Associations between self-rated health and depressive symptoms among older adults: does age matter? Soc. Sci. Med. 280:114024. doi: 10.1016/j.socscimed.2021.114024, 34049050

[ref31] Radloff L. S. (1977). The CES-D scale: a self-report depression scale for research in the general population. Appl. Psychol. Meas. 1, 385–401. doi: 10.1177/014662167700100306

[ref32] Santabárbara J. Lipnicki D. M. Villagrasa B. Lobo E. Lopez-Anton R. (2019). Anxiety and risk of dementia: systematic review and meta-analysis of prospective cohort studies. Maturitas 119, 14–20. doi: 10.1016/j.maturitas.2018.10.014, 30502746

[ref33] Shi Z. L. Zhu Z. J. Zhang H. Cao W. N. Ji Y. Shi Y. H. (2025). Prevalence and influencing factors of cognitive impairment among Chinese older adults: based on CHARLS data. Chin. J. Gerontol. 45, 1511–1514. doi: 10.1007/s10339-025-01301-9

[ref34] Sommerlad A. Ruegger J. Singh-Manoux A. Lewis G. Livingston G. (2018). Marriage and risk of dementia: systematic review and meta-analysis of observational studies. J. Neurol. Neurosurg. Psychiatry 89, 231–238. doi: 10.1136/jnnp-2017-316274, 29183957 PMC5869449

[ref35] Su Y. J. Sun Y. Ling Y. Zhang K. Liu X. Y. Peng G. P. (2023). Correlation between depression and cognitive function in elderly patients with mild cognitive impairment. Chin. J. Contemp. Neurol. Neurosurg. 23, 292–302. doi: 10.1080/10615806.2023.2255531

[ref36] Sun Y. C. Feng J. Lei Z. H. Qu G. Li X. Y. Gan Y. (2024). Relationship between depressive symptoms and cognitive function among middle-aged and elderly people in China. Chin. J. Public Health 40, 1206–1211. doi: 10.1007/s00406-024-01854-4

[ref37] Tang J. (2022). Global and China’s population aging: causes and implications. Soc. Policy Res. 3–18. doi: 10.19506/j.cnki.cn10-1428/d.2022.03.008

[ref38] Tu A. X. Liu X. Jiang M. M. (2024). Cognitive function status of the elderly and the influence of personality traits. Chin. Health Serv. Manag. 41, 940–944. doi: 10.1038/s41598-024-78483-3

[ref39] Virgili G. Parravano M. Petri D. Maurutto E. Menchini F. Lanzetta P. . (2022). The association between vision impairment and depression: a systematic review of population-based studies. J. Clin. Med. 11:2412. doi: 10.3390/jcm11092412, 35566537 PMC9103717

[ref40] Wang A.-Y. Hu H.-Y. Ou Y.-N. Wang Z.-T. Ma Y.-H. Tan L. . (2023). Socioeconomic status and risks of cognitive impairment and dementia: a systematic review and Meta-analysis of 39 prospective studies. J. Prev Alzheimers Dis. 10, 83–94. doi: 10.14283/jpad.2022.81, 36641612

[ref41] Wang G. Qi J. L. Liu X. Y. Ren R. J. Lin S. H. Hu Y. S. . (2024). China Alzheimer Report 2024. J. Diagnost. Concepts Pract. 23, 219–256. doi: 10.16150/j.1671-2870.2024.03.001

[ref42] Weden M. M. Shih R. A. Kabeto M. U. Langa K. M. (2018). Secular trends in dementia and cognitive impairment of U.S. rural and urban older adults. Am. J. Prev. Med. 54, 164–172. doi: 10.1016/j.amepre.2017.10.021, 29246677 PMC5783777

[ref43] Weidner W. S. (2023). World Alzheimer report 2022 - how strong are global health systems: lessons learned and case studies from across the globe. Alzheimers Dement. 19:e073714. doi: 10.1002/alz.073714

[ref44] Xia X. Jiang Q. McDermott J. Han J.-D. J. (2018). Aging and Alzheimer’s disease: comparison and associations from molecular to system level. Aging Cell 17:e12802. doi: 10.1111/acel.12802, 29963744 PMC6156542

[ref45] Xinhua News Agency (2024). Study the “Decision” Q&A: How to Understand Proactively Responding to Population Aging and Improving the Policy Mechanisms for Elderly care Services and Industries. Beijing, China: Central People’s Government of the People’s Republic of China. Available online at: https://www.gov.cn/zhengce/202410/content_6983026.htm (Accessed April 18, 2026).

[ref46] Ye S. C. Wang J. (2025). Research progress on influencing factors of cognitive impairment in elderly depression. Hainan Med. J. 36, 1061–1064. doi: 10.31577/cai_2025_4_961

